# Fabrication of Novel MOF/HOF Composite for Efficient Degradation of Methylene Blue via Photo-Fenton-like Process

**DOI:** 10.3390/molecules30244691

**Published:** 2025-12-08

**Authors:** Yanfeng Zhang, Yong Huang, Han Leng, Xuwei Chen

**Affiliations:** 1 Intelligent Policing Key Laboratory of Sichuan Province, Sichuan Police College, Luzhou 646000, China; zhyf@scpolicec.edu.cn; 2 Department of Chemistry, College of Sciences, Northeastern University, Box 332, Shenyang 110819, China; chbu314@163.com (Y.H.); lengh@mails.neu.edu.cn (H.L.)

**Keywords:** photo-Fenton process, MOF@HOF composite, methylene blue, pollutant degradation

## Abstract

The photo-Fenton process is an advanced oxidation method widely employed in environmental remediation. Herein, we developed a novel metal–organic framework@hydrogen-bonded organic framework (MOF/HOF) composite with excellent photo-Fenton-like activity for the efficient degradation of organic dye methylene blue (MB). Cu-based MOF (CuBTC) was firstly prepared via the solvothermal method, then melamine (MA) and trimesic acid (TMA)-based HOF (MA-TMA) was grown in situ on CuBTC with hydrogen bonding interactions to produce the MOF/HOF composite CuBTC-MA. The CuBTC-MA composite could catalyze H_2_O_2_ to produce active substances for efficient MB degradation. The degradation rate constant of the CuBTC-MA composite was 4.4 times and 16.7 times higher than that of CuBTC and MA-TMA. The remarkably enhanced performance was attributed to the synergistic effect between the efficient separation of electron–holes supported by the type-II heterojunction structure of the CuBTC-MA composite and the Cu(I)/Cu(II) inter-conversion. The CuBTC-MA composite demonstrated exceptional repeatability and maintained a stable performance across a broad pH range. This study provided a novel paradigm for engineering heterogeneous MOF/HOF heterostructures, demonstrating significant potential in advancing photo-Fenton-like catalytic systems for the efficient environmental remediation of organic pollutants through synergistic charge separation and radical generation mechanisms.

## 1. Introduction

Rapid industrialization has led to the substantial discharge of dye-containing wastewater, posing significant risks to ecosystem stability and human health. To address this challenge, extensive research efforts have focused on developing advanced wastewater treatment technologies, including adsorption, photocatalysis, biodegradation, and advanced oxidation processes (AOPs) [1]. Among these, Fenton-based oxidation systems have emerged as promising solutions for degrading recalcitrant pollutants and persistent organic contaminants through hydroxyl radical generation [2]. However, critical limitations persist in conventional Fenton applications, including a narrow operational pH range (3–5), excessive H_2_O_2_ consumption, and the generation of iron hydroxide [3]. These constraints highlight the urgent need for developing efficient Fe-free heterogeneous photocatalytic systems that combine environmental compatibility with operational practicality.

Metal–organic frameworks (MOFs) and hydrogen-bonded organic frameworks (HOFs) are periodic crystalline porous materials assembled via coordination bonds and hydrogen bonding interactions, respectively. These materials exhibit notable advantages including a high porosity, adjustable pore size, large specific surface area, lattice stability, and abundant catalytic sites [4], and are widely employed in gas storage [5,6], chemical sensing [7,8], adsorption [9,10], drug delivery [11,12], heterogeneous catalysis [13,14], and other fields. HOFs exhibit limited application in photo-Fenton-like catalysis due to their inherent instability and suboptimal catalytic activity. Recent advances demonstrate that integrating HOFs with MOFs as matrix substrates enables robust in situ assembly and the multi-site growth of HOF/MOF heterostructures, significantly enhancing catalytic performance [15]. MOFs have emerged as promising photocatalysts for pollutant degradation via photo-Fenton-like mechanisms, e.g., Fan et al. reported the fabrication of Bi-MOF-based solar evaporators for photo-Fenton Cr(VI) reduction. When integrating with a photo-Fenton-like reaction, the obtained reaction kinetics (0.0548 min^−1^) of Bi-MOF surpassed many advanced catalysts [16]. Guan et al. demonstrated that a Fe-doped MOF exhibited a degradation efficiency of 97% to bisphenol A within 30 min, and the reaction rate constant was high up to 0.1209 min^−1^ [17]. Li et al. indicated that MOF-on-MOF derivatives could serve as promising photo-Fenton catalysts for ciprofloxacin removal, with approximately 99.1% degradation of CIP within 120 min under neutral conditions [18]. It should be noted that monolithic MOF materials face critical challenges including wide bandgaps, restricted visible light absorption, rapid electron–hole recombination, and inefficient charge transport. To address these limitations, researchers have developed multifunctional strategies including element doping [19], defect engineering [20], and the formation of heterojunctions [21,22,23] to improve the activity of the catalyst. The formation of heterojunction interfaces enables efficient interfacial charge transfer and the spatial separation of photogenerated electron–hole pairs, effectively suppressing recombination processes while prolonging carrier lifetimes [24]. This mechanism significantly enhances photo-Fenton catalytic efficiency through sustained reactive oxygen species (ROS) generation. Although Fe-based metal–organic frameworks (Fe-MOFs) dominate current photo-Fenton research, they inherit limitations from conventional Fenton systems, including narrow pH operational windows and rapid Fe(II)/Fe(III) redox cycling [25]. Cu-based MOFs (Cu-MOFs) exhibit analogous redox activity with extended pH adaptability, positioning them as promising alternatives. Meanwhile, the engineering of Cu-MOFs/HOFs heterostructured composites with optimized interfacial charge transfer pathways can significantly enhance photo-Fenton-like catalytic efficiency.

In the present study, a novel Cu-based MOF/HOF composite (CuBTC-MA) was fabricated and employed as a photo-Fenton catalyst for methylene blue (MB) degradation. The Cu-based MOF (CuBTC) was prepared via the solvent thermal procedure, then the HOF material MA-TMA was grown in situ on CuBTC based on the H-bond interaction between homophenic acid and melamine (MA). CuBTC-MA was used for the photo-Fenton-like degradation and removal of MB. The structure of CuBTC-MA was similar to a type-II heterojunction, which facilitated efficient interfacial charge transfer through the separation of photogenerated electron–hole pairs, while Cu(I)/Cu(II) redox cycling enabled sustained Fenton-like radical generation. The influence of parameters affecting the photo-Fenton-like degradation reaction were systematically investigated, and the stability and reusability of the CuBTC-MA composite were evaluated. Under the optimal conditions, the photo-Fenton-like performance of CuBTC-MA was enhanced greatly when compared with the single CuBTC and MA-TMA. Meanwhile, based on the conduction band and valence band position of the material and the capture performance of active free radicals, the photo-Fenton-like reaction mechanism of CuBTC-MA composite was explored.

## 2. Results and Discussion

### 2.1. Fabrication and Characterizations of CuBTC-MA Composite

**Scheme 1:**
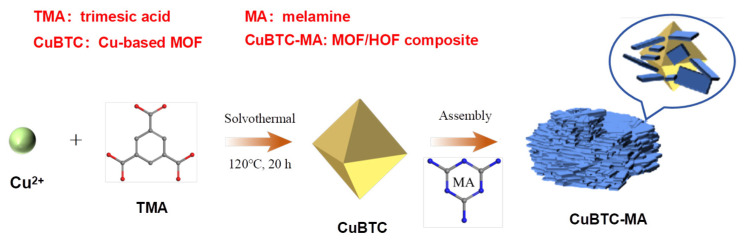
Schematic diagram for the fabrication of CuBTC-MA composite.

The preparation process of the CuBTC-MA composite is illustrated in Scheme 1. Cu-based MOF CuBTC was firstly prepared by the self-assembly of a Cu^2+^ metal center and the organic ligand H_3_BTC under solvothermal conditions. Thereafter, hydrogen-bonded organic framework MA-TMA was grown in situ on a CuBTC surface under H-bonding interaction conditions between H_3_BTC and MA.

The morphologies of the CuBTC, MA-TMA, and CuBTC-MA composites were observed using scanning electron microscope (SEM) and transmission electron microscope (TEM). As shown in Figure S1A–C, CuBTC and MA-TMA presented aggregated nanoparticles and random block shapes, respectively, while the CuBTC-MA composite demonstrated a hierarchical architecture formed through a stacked layered structure of MA-TMA encapsulating CuBTC nanoparticles (Figure S1D). Energy-dispersive spectrometer (EDS) results revealed that Cu, C, N, and O elements were uniformly distributed in the CuBTC-MA composite (Figure S1E).

**Figure 1:**
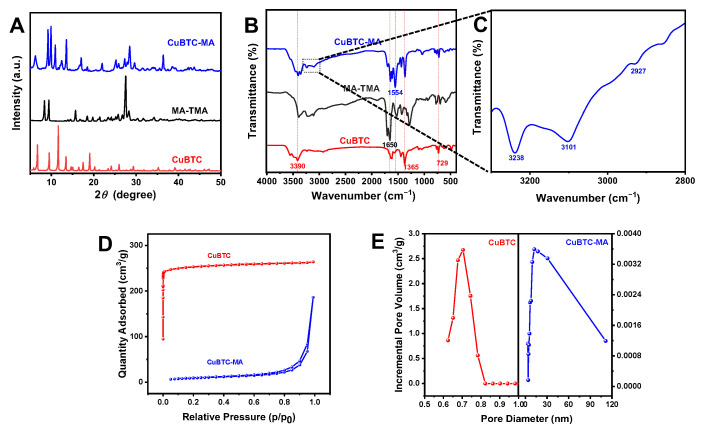
(**A**) PXRD patterns and (**B**) FT-IR spectra of CuBTC, MA-TMA, and CuBTC-MA composites. (**C**) Local magnified FT-IR spectra of CuBTC-MA in range of 2800~3300 cm^−1^. (**D**) N_2_ adsorption/desorption isotherms and (**E**) pore size distributions of CuBTC and CuBTC-MA composites.

Figure 1A shows the X-ray diffraction (XRD) patterns of the CuBTC, MA-TMA, and CuBTC-MA composites. The diffraction peaks of CuBTC appeared at 2θ = 6.7°, 9.5°, 11.6°, and 13.5°, which belonged to the reflections of crystal planes (200), (220), (222), and (400) [26]. The series of diffraction peaks of MA-TMA at 2θ = 8.4°, 9.4°, 15.7°, and 27.6° belonged to the reflections of crystal planes (100), (110), (210), and (112), which were consistent with that of previously reported work [27]. It could be seen that the CuBTC-MA composite exhibited the characteristic peaks of both CuBTC and MA-TMA. The diffraction peak at 11.6° in the original CuBTC shifted slightly to 11.0°, due to the interaction between CuBTC and MA-TMA.

As illustrated in Figure 1B, the FT-IR spectrum of CuBTC exhibited characteristic peaks at 1650 cm^−1^ and 1356 cm^−1^, attributed to the C=O and C=C stretching vibrations of the H_3_BTC ligand, respectively. The peak at 729 cm^−1^ could be assigned to the Cu-O stretching vibration [26]. In the FT-IR spectrum of MA-TMA, the peaks at 3390 cm^−1^ and 1554 cm^−1^ were ascribed to the –N-H stretching vibration and –C=N stretching vibration, respectively. As for the CuBTC-MA composite, the peaks from both CuBTC and MA-TMA confirmed the coexistence of these components within the composite structure, and the characteristics of intermolecular hydrogen bonds (O···H-N or N···H-N) [15] were clearly observed at 3238 cm^−1^, 3101 cm^−1^, and 2927 cm^−1^ (Figure 1C).

The N_2_ adsorption–desorption isotherms and pore size distributions of the CuBTC and CuBTC-MA composites are shown in Figure 1D,E. CuBTC presented a type-I adsorption isotherm, while the CuBTC-MA composite displayed a type-IV isotherm, which is a typical feature of mesoporous materials. The specific surface area of the CuBTC-MA composite acquired via the Brunauer–Emmett–Teller (BET) determination was 32.51 m^2^/g, with an average pore size of 12.348 nm. The mesoporous structure was conducive to enhancing the diffusion and transport of reactants, thereby improving the photo-Fenton-like activity of the CuBTC-MA composite. The BET surface area of pristine CuBTC was 1049.44 m^2^/g, while MA-TMA exhibited a negligible surface area (10.27 m^2^/g) due to its non-porous nature. Compared with pristine CuBTC, the CuBTC-MA composite showed a significant reduction in surface area. This sharp decrease might be attributed to the partial blockage of CuBTC pores by MA molecules during the encapsulation process, limiting nitrogen accessibility and reducing effective porosity. Additionally, the formation of hydrogen bonds between CuBTC and MA may lead to structural deformation, further diminishing the available surface area.

**Figure 2:**
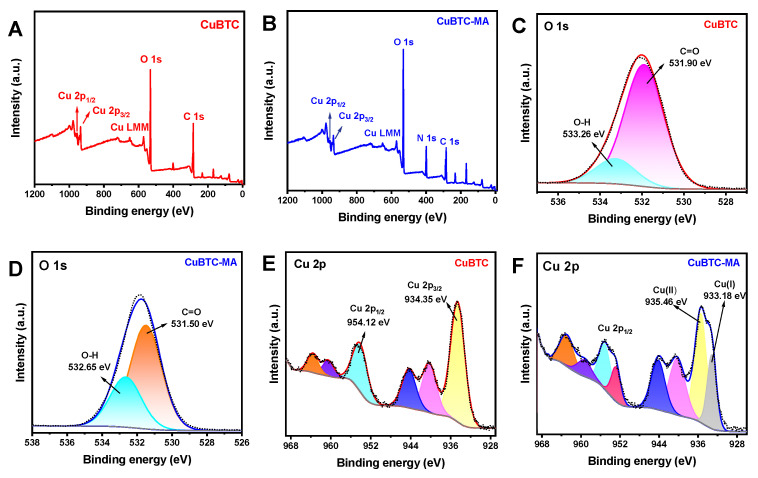
XPS spectra of CuBTC (**A**) and CuBTC-MA composites (**B**). High-resolution O 1s spectra of CuBTC (**C**) and CuBTC-MA (**D**). High-resolution Cu 2p spectra of CuBTC (**E**) and CuBTC-MA (**F**).

To elucidate the chemical composition of the CuBTC and CuBTC-MA composites, X-ray photoelectron spectroscopy (XPS) investigations were performed. As illustrated in Figure 2A, the peaks of C 1s, O 1s, and Cu 2p can be evidently observed in the XPS spectra of CuBTC, and the appearance of N 1s in the CuBTC-MA composite (Figure 2B) demonstrates well the growth of HOF on MOF. In the high-resolution O 1s spectrum of CuBTC, the peaks at 531.90 eV and 533.26 eV were attributed to O-H and C=O, respectively (Figure 2C). These two peaks shifted to 531.50 eV and 532.65 eV in CuBTC-MA (Figure 2D), due to the formation of H-bonds. In the high-resolution Cu 2p spectra of CuBTC (Figure 2E), the characteristic peaks at 954.12 eV and 934.35 eV represented the Cu 2p1/2 and Cu 2p3/2 orbits, indicating the existence of Cu(II) in the material. It should be noted that the CuBTC-MA composite exhibited a new peak at 933.18 eV (Figure 2F), along with the shift in Cu 2p3/2 peak to 935.46 eV, suggesting the partial reduction of Cu(II) to Cu(I).

### 2.2. Photo-Fenton-like Degradation of MB with CuBTC-MA Composite

**Figure 3:**
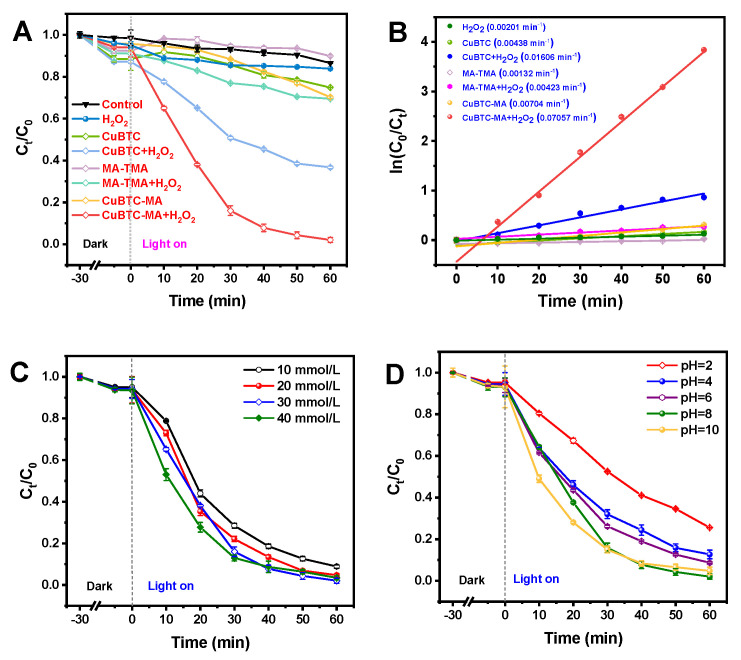
The photo-Fenton-like degradation curves (**A**) and kinetic fitting curves (**B**) of MB under different catalysis systems. The photo-Fenton-like curves of MB under different H_2_O_2_ concentrations (**C**) and pH conditions (**D**).

The photo-Fenton-like catalytic activities of the CuBTC, MA-TM, and CuBTC-MA composites for MB degradation in the absence/presence of H_2_O_2_ were systematically investigated. As shown in Figure 3A, MB itself exhibited nearly no degradation under light irradiation, and poor MB degradation was observed for the CuBTC, MA-TM, and CuBTC-MA composites in the absence of H_2_O_2_. In the presence of H_2_O_2_, the CuBTC-MA composite demonstrated a superior photocatalytic performance for MB degradation, achieving a 97.8% degradation efficiency within 60 min, which was much higher than that of CuBTC (57.8%) and MA-TMA (21.2%). The obvious improved catalytic activity of CuBTC-MA demonstrated well the critical role of HOF growth on CuBTC. The kinetic analysis results revealed that the MB degradation behaviors under the CuBTC-MA composite (Figure 3B) conformed to first-order degradation patterns [28]. Notably, the dynamic rate constant (k) of the photo-Fenton-like reaction for the CuBTC-MA composite in the presence of H_2_O_2_ was 0.07057 min^−1^, which was improved by 4.4-fold and 16.7-fold compared with that of CuBTC (0.01606 min^−1^) and MA-TMA (0.00423 min^−1^). Table 1 summarizes the photo-Fenton-like catalytic performances of the reported materials for MB degradation. Compared with the Fe_3_O_4_-based catalysts, non-metallic g-C_3_N_4_, and Fe-doped bimetallic composites, the CuBTC-MA composite offered the highest dynamic rate constant under the comparable degradation time condition due to the improved separation of photogenerated electrons and holes, suggesting the great potential of the CuBTC-MA composite in practical applications.

In order to achieve the best photo-Fenton-like catalytic performance of the CuBTC-MA composite, the effect of H_2_O_2_ concentration on MB degradation was studied. MB degradation efficiency firstly increased slightly with H_2_O_2_ concentration (Figure 3C), then declined when the H_2_O_2_ concentration was higher than 30 mmol/L. The decline in the MB degradation efficiency at a high H_2_O_2_ concentration might be attributed to the fact that the excessive H_2_O_2_ would induce ·OH depletion through a self-quenching reaction (·OH + H_2_O_2_ → ·HO_2_ + H_2_O), thereby diminishing the oxidation capacity of the catalytic system [40].

Figure 3D illustrates the degradation behaviors of MB in the pH range of 2–10. It can be seen that the best degradation efficiency was achieved at pH 8, and an obvious decline in the degradation efficiency took place when the degradation was performed under pH 2. The reason might lie in the fact that the protonation of CuBTC-MA under strong acidic conditions made the catalyst’s surface positively charged, while the electrostatic repulsion between CuBTC-MA and cationic dye prevented the adsorption of MB onto the composite, resulting in the poor degradation efficiency. It should be noted that favorable MB degradation was achieved in the pH range of 4–10, suggesting the potential practicability of MB in handling real samples.

### 2.3. Reusability of CuBTC-MA Composite

**Figure 4:**
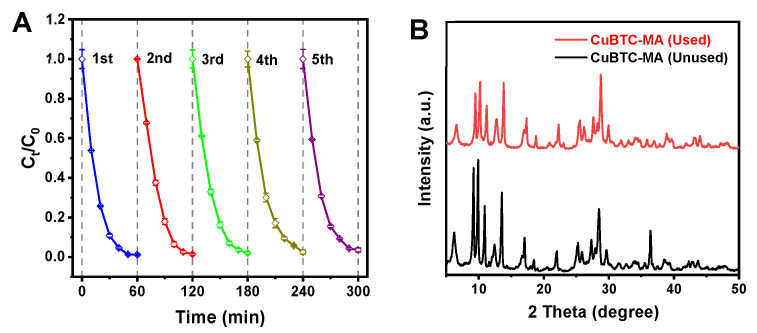
(**A**) Reusability of CuBTC-MA composite for MB degradation. (**B**) XRD patterns of CuBTC-MA composite before and after photo-degradation process.

To assess the practical potential of the CuBTC-MA composite for MB degradation, the CuBTC-MA composite after MB degradation was collected and regenerated using ethanol after washing with deionized water. As illustrated in Figure 4A, nearly no deterioration in the degradation efficiency was observed after repeated usage. The degradation efficiency remained higher than 96.5% after five cycles, suggesting the excellent reusability of the CuBTC-MA composite in the photo-Fenton-like degradation process. Moreover, comparative XRD patterns revealed that no structural alteration was observed between the fresh and regenerated CuBTC-MA composite, confirming structural integrity during repeated usage (Figure 4B).

### 2.4. Photocatalytic Mechanism of CuBTC-MA Composite

**Figure 5:**
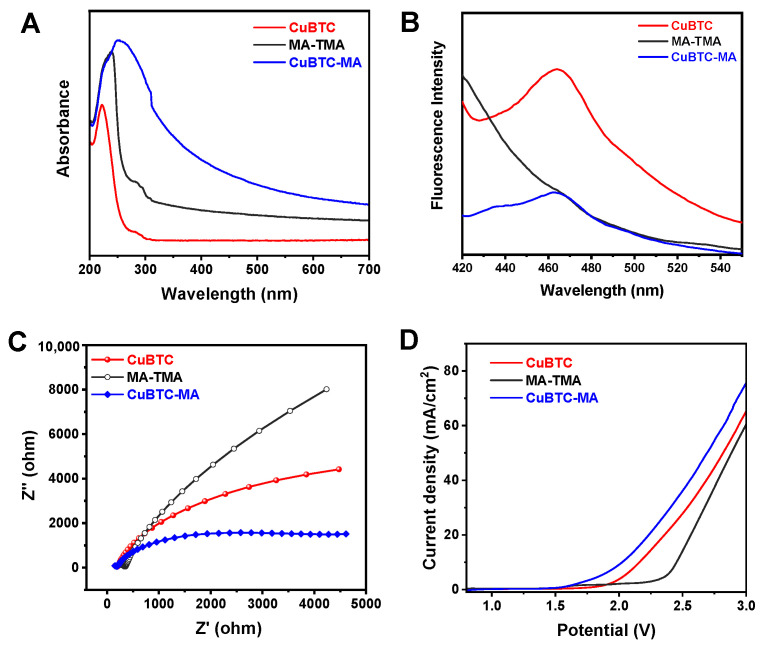
(**A**) UV-vis absorption spectra, (**B**) steady-state photoluminescence spectra, (**C**) EIS Nyquist plots, and LSV curves (**D**) of CuBTC, MA-TMA, and CuBTC-MA composites.

The optical properties of the material itself might greatly affect its photo-Fenton-like activity; the absorption characteristics of the CuBTC, MA-TMA, and CuBTC-MA composites were thus studied using UV-vis absorption diffuse reflection spectroscopy. As illustrated in Figure 5A, compared with CuBTC and MA-TMA, the CuBTC-MA composite exhibited stronger absorption. Moreover, the absorption band edge extended to the near-infrared region, suggesting the superior light absorption capability of the CuBTC-MA composite. Based on the Kubelka–Munk curves (Figure S2A), the bandgap widths (Eg) of CuBTC and MA-TMA were calculated to be 3.50 eV and 4.30 eV, respectively, based on Tauc plots [41]. Steady-state photoluminescence (PL) spectra of the CuBTC, MA-TMA, and CuBTC-MA composites were recorded and used to evaluate the charge separation efficiency of these materials, as a strong PL response corresponds to a high electron–hole recombination probability [42]. Both the CuBTC and CuBTC-MA composites shared a similar emission maximum (Figure 5B), while the PL intensity of the CuBTC-MA composite was significantly lower than that of CuBTC, indicating the reduced charge recombination rates and improved photo-Fenton-like catalytic activity of the CuBTC-MA composite.

To achieve the valence band (VB) positions of CuBTC and MA-TMA, an XPS valence band analysis was performed. Based on the energy distribution curve derived from the acquired VB spectra (Figure S2B), the VB potentials of CuBTC and CuBTC-MA were 1.85 eV and 2.72 eV, respectively [43]. According to the relationship of Eg = E_VB_ − E_CB_ [44], the conduction band (CB) potentials of CuBTC and CuBTC-MA were deduced to be −1.65 eV and −1.58 eV, respectively.

Electrochemical impedance spectroscopy (EIS) was employed to assess the charge transfer resistance of the CuBTC, MA-TMA, and CuBTC-MA composites during the charge transfer process, and the obtained Nyquist curves are illustrated in Figure 5C. Compared with CuBTC and MA-TMA, the CuBTC-MA composite exhibited the smallest arc radius, indicating the lowest charge transfer resistance and the highest separation efficiency of photogenerated electron–hole pairs. Figure 5D shows the linear sweep voltammetry (LSV) curves of the CuBTC, MA-TMA, and CuBTC-MA composites at a scan rate of 100 mV s^−1^ under visible light illumination. CuBTC-MA presented a higher photocurrent density than CuBTC and MA-TMA, suggesting enhanced photo-excited charge carrier generation and transport under illumination.

To identify the primary active oxygen species (ROS) involved in MB degradation, radical trapping investigations were conducted using isopropanol (IPA), ascorbic acid (AA), L-histidine (L-his), and disodium ethylenediaminetetraacetate (EDTA-2Na) as the specific scavengers for ·OH, ·O_2_^−^, ^1^O_2_, and *h^+^* respectively. As illustrated in Figure S3A, the presence of L-his nearly did not affect the degradation behaviors of MB. A certain decrease in the degradation efficiency was observed for AA, while the degradation of MB was significantly reduced upon the introduction of IPA and EDTA-2Na. These results strongly suggested that ·OH and *h^+^*, as well as ·O_2_^−^, were the main radical species playing critical roles in the photo-Fenton-like degradation of MB by the CuBTC-MA composite. Electron paramagnetic resonance (ESR) spectroscopy was employed to validate the ·OH generation with 5,5-dimethyl-1-pyrrolidine N-oxide (DMPO) serving as the hydroxyl radical trapping reagent [45]. Nearly no signal was observed for MA-TMA, and the CuBTC-MA composite exhibited a much higher ESR intensity than CuBTC (Figure S3B). The ·OH generation in the CuBTC-MA/H_2_O_2_ system was further confirmed using a phthalic acid (TPA)-based photoluminescence analysis [22]. TPA can react with ·OH to produce fluorescent 2-hydroxyterephthalic acid (TAOH). As shown in Figure S3C, the CuBTC-MA composite provided the highest photoluminescence response, indicating the superior ·OH production capability of the CuBTC-MA composite.

### 2.5. Mechanism for the Photo-Fenton-like Degradation of MB

**Figure 6:**
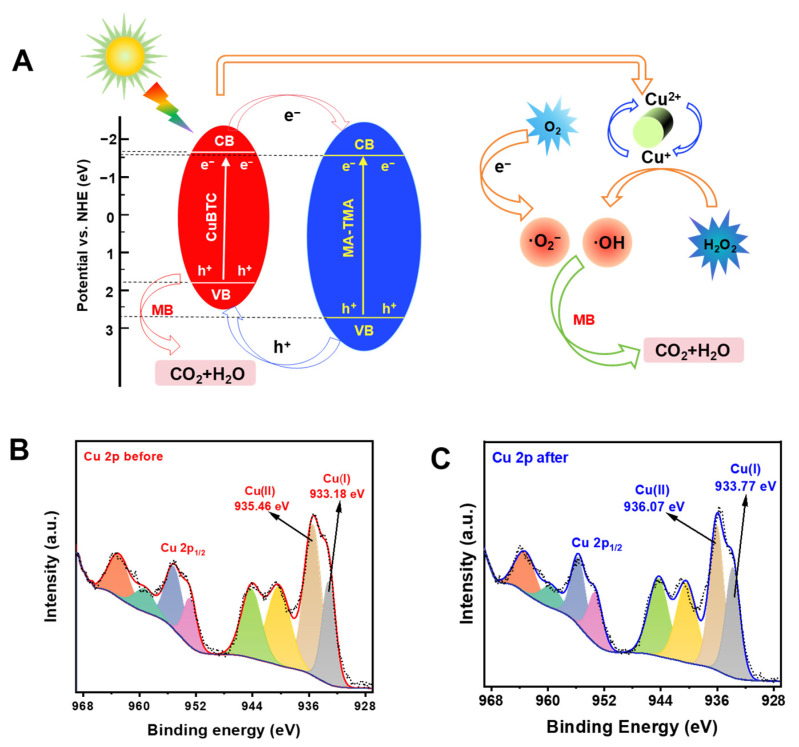
(**A**) Mechanism of CuBTC-MA photo-degradation toward MB. Cu 2p XPS spectra of CuBTC-MA (**B**) before and (**C**) after photocatalytic reaction.

Based on the CB and VB potentials of CuBTC and MA-TMA, it could be inferred that a type-II heterojunction structure was formed in the CuBTC-MA composite, thus the possible mechanism for MB degradation by a CuBTC-MA photo-Fenton-like system was proposed. As illustrated in Figure 6A, the photogenerated electrons in the valence bands of both components of CuBTC-MA were excited to the conduction band under visible light irradiation, leaving *h^+^* in the valence bands. Due to the more negative CB potential and smaller VB potential of CuBTC, the photogenerated electrons migrated rapidly from CuBTC’s conduction band to MA-TMA’s conduction band, and subsequently reached the surface to reduce O_2_ into ·O_2_^−^. At the same time, *h^+^* transferred from MA-TMA’s valence band to CuBTC’s valence band, facilitating MB degradation along with ·O_2_^−^. Meanwhile, Cu (II) leaked from CuBTC-MA participated in the photo-Fenton-like process by reacting with H_2_O_2_ to generate ·O_2_^−^, while the produced Cu(I) was subsequently reoxidized into Cu(II) by H_2_O_2_, concomitantly generating ·OH through Fenton-like redox cycling as described by the following processes [46].

Cu(II) + H_2_O_2_ → Cu(I) + ·O_2_^−^ + 2H^+^ (1)

Cu(I) + H_2_O_2_ → Cu(II) + ·OH + OH^−^ (2)

·O_2_^−^/·OH/h^+^ + MB → degradation products (3)

To verify that the interfacial electron transfer in the composite followed type-II heterojunction behavior, XPS analyses on Cu 2p spectra were conducted. As illustrated in Figure 6B,C, the Cu 2p binding energy of Cu(II) and Cu(I) both shifted significantly to high fields, which indicated that the photogenerated electron immigrated from component CuBTC to MA-TMA, confirming that the electron transfer path was similar to a type-II heterojunction.

### 2.6. Possible Degradation Pathways of MB

To explore the degradation pathways of MB, the structure of MB was simulated using Gaussian software, and the Fukui indexes of MB molecule are listed in Table S1. The results revealed that the positive charge was mainly distributed at 19N, 30N, and 29S of MB (Figure S4A), and HOMO-LUMO distributions revealed preferential localization on MB’s phenothiazine ring and dimethyl nitrogen (Figure S4B), rendering these regions vulnerable to free radical attack by ·OH species. The HOMO-LUMO analysis was limited in precisely identifying electron transfer sites; therefore, two possible degradation pathways of MB were proposed based on the obtained Fukui indexes. For pathway I, the 19N of high ƒ^0^ (0.0612) was attacked by ·OH, leading to the N-dealkylation of MB to give the intermediates P1 (*m*/*z* = 270) and P2 (*m*/*z* = 256). The similar attack on 30N resulted in the intermediate P3 (*m*/*z* = 228). Subsequently, the breakage of C=N and C-S bonds under radical attack led to the disruption of the phenothiazine ring, giving the intermediate P4 (*m*/*z* = 188). Pathway II firstly involved the oxidation of 29S to form the intermediate P5 (*m*/*z* = 301), then the dealkylation of 18N and 30N in turn to give intermediates P6 (*m*/*z* = 273) and P7 (*m*/*z* = 245). Thereafter, the disruption of the phenothiazine ring gave the intermediates P8 (*m*/*z* = 150), P9 (*m*/*z* = 165), P10 (*m*/*z* = 221), and P11 (*m*/*z* = 74). Finally, the resulting intermediates P4, P8, P9, P10, and P11 underwent a series of successive degradations to produce CO_2_ and H_2_O (Figure S5). All calculations were obtained using Gaussian 16 and Multiwfn 3.8 software and visualized using VMD 1.9.3 [47].

## 3. Materials and Methods

### 3.1. Chemicals and Reagents

Cu(NO_3_)_2_·3H_2_O, methylene blue (MB), melamine (MA), hydrochloric acid, phosphoric acid, boric acid, glacial acetic acid, anhydrous ethanol, N, N-dimethylformamide (DMF), p-benzoquinone (p-BQ), and disodium ethylenediaminetetraacetic acid (EDTA-2Na) were purchased from Sinopharm Chemical Reagent Co., Ltd. (Shanghai, China). 1,3,5-phenyltrimethoic acid (H_3_BTC) was purchased from Aladdin Reagent Co., Ltd. (Shanghai, China). Sodium hydroxide was purchased from Damao Chemical Reagent Factory (Tianjin, China). All reagents were analytical reagent grade and used as received without further purification. Deionized (DI) water (18 MΩ/cm) was used throughout all experiments.

### 3.2. Preparation of Cu-Based MOF CuBTC

CuBTC was prepared based on a reported solvothermal method with minor modification [26]. Typically, Cu(NO_3_)_2_·3H_2_O (8.07 mmol, 1.95 g) and H_3_BTC (4.76 mmol, 1.00 g) were dissolved into 50 mL of a mixed solvent system (H_2_O:EtOH:DMF = 1:1:1, *v*/*v*/*v*). The resultant mixture underwent magnetic stirring for 30 min to form a homogeneous suspension, and then heated at 120 °C for 24 h under a controlled atmosphere. After cooling to room temperature, the mixture was directed to centrifugation at 8000 rpm for 5 min. The collected product was washed sequentially with ethanol and deionized water in turn, then dried under vacuum at 60 °C for 12 h. The blue product was named CuBTC.

### 3.3. Synthesis of HOF MA-TMA

HOF material MA-TMA was synthesized according to an established protocol [27]. MA (0.5 mmol, 0.0631 g) and H_3_BTC (0.5 mmol, 0.1051 g) were dissolved in 30 mL deionized water, then mixed together and stirred at room temperature for 1 h. The precipitate was collected after centrifugation, washed with deionized water, and dried under vacuum at 60 °C for 12, to give the white product of MA-TMA.

### 3.4. Fabrication of MOF@HOF Composite CuBTC-MA

Briefly, MA (0.40 mmol, 50.00 mg) was dissolved in 30 mL deionized water under magnetic stirring at 80 °C. Subsequently, 100 mg of CuBTC was introduced into the MA solution, and the resultant mixture was stirred at room temperature for 1 h. The produced precipitate was collected via centrifugation, washed with deionized water, and dried under vacuum at 60 °C for 12 h; the final light blue product was the CuBTC-MA composite.

### 3.5. Characterizations of CuBTC-MA Composite

Hitachi SU-8000 scanning electron microscopy (Hitachi, Tokyo, Japan) and JEOL JEM-2100PLUS transmission electron microscopy (JEOL USA, Peabody, MA, USA) were employed to characterize the morphology of the materials. Powder X-ray diffraction (PXRD) patterns were collected on a Maxima XRD-7000 diffractometer (Shimadzu, Kyoto, Japan). N2 adsorption–desorption isotherms were calculated by an Autosorb-IQ-MP-C automatic gas adsorption analyzer at 77 K (Quantachrome, FLA, USA). Fourier transform infrared (FT-IR) spectra were recorded using a Nicolet-6700 FT-IR spectrophotometer (Thermo Fisher Scientific, Waltham, MA, USA). Thermo Scientific ESCALAB 250 Xi X-ray Photoelectron Spectrometer using 1486.6 eV Al Kα radiation (Thermo Fisher Scientific, Waltham, MA, USA) was employed to measure X-ray photoelectron spectroscopy (XPS) spectra. Ultraviolet–visible (UV-vis) absorption spectra were obtained on a U-3600 UV-vis spectrophotometer (Hitachi, Tokyo, Japan).

### 3.6. Evaluation of Photo-Fenton-like Catalytic Capacity

The photo-Fenton-like catalytic ability of the CuBTC-MA composite was systematically evaluated by monitoring the MB degradation kinetics. In the quartz photochemical reactor of a homemade photo-Fenton device (Figure S6), 1 mg CuBTC-MA composite was added into 10 mL MB solution (10 mg/L, prepared in BR buffer of pH 3.0) and magnetically stirred in the dark to reach adsorption equilibrium. Subsequently, the reaction system was illuminated under a halogen lamp (300 W) with a 20 cm irradiation distance. Thereafter, 30 μL H_2_O_2_ (9.8 mmol/L) was introduced to initiate the photocatalytic reaction. During the photocatalytic process, the temperature of the reaction system was maintained at 25 °C via circulating water. Then 100 μL of the supernatant was extracted every 10 min, while the absorbance at 664 nm was measured and used to determine the concentration of residual MB.

The degradation efficiency of MB was calculated according to following equation.

Degradation efficiency (%) = *C*_t_/*C*_0_ × 100% (4)

where *C*_0_ and *C*_t_ represent the original concentration and the residual concentration of MB at each time point.

The photo-Fenton-like degradation ability of the CuBTC-MA composite was evaluated according to a first-order kinetic model [45], and the dynamic rate constant k was calculated via following equation.

Ln (*C*_0_/*C*_t_) = kt (5)

where k is the dynamic rate constant and t represents the reaction time (min).

## 4. Conclusions

In summary, a novel heterogeneous photo-Fenton-like catalyst, a CuBTC-MA composite, was fabricated via the in situ growth of HOF MA-TMA on Cu-based MOF CuBTC via hydrogen bonding interactions, and the performance of the CuBTC-MA composite for the photo-Fenton-like degradation of methylene blue was investigated systematically. The as-fabricated HOF@MOF composite exhibited a much higher degradation rate constant than the pristine MOF and HOF components. The enhanced photo-Fenton-like activity of the CuBTC-MA composite lies in the fact that the formed type-II-like heterojunction structure between the MOF and HOF components improved the separation of photogenerated electrons and holes significantly. Meanwhile, Cu(I)/Cu(II) redox cycling in the material also facilitated the photo-Fenton-like catalytic reaction. Notably, the CuBTC-MA composite showed excellent reusability and a stable photo-Fenton-like catalytic capacity across a wide pH range, making it a substantial candidate for the efficient degradation of organic dye pollutants. This research not only provides a new photo-Fenton-like catalytic material as a promising platform for environmental remediation, but also highlights the potential of MOFs in the catalysis field via fabricating powerful composites of heterojunction structures.

## Figures and Tables

**Table 1:** Catalytic performance of various photocatalysts in photo-Fenton-like degradation of MB.

Catalysts	Catalyst Conc. (g/L)	MB Conc. (mg/L)	H_2_O_2_ Conc. (mmol/L)	Time (min)	Degradation Efficiency (%)	K (min^−1^)	Ref.
Fe_3_O_4_@C	0.8	10	90	60	100	-	[29]
Fe_3_O_4_@GO	0.003	5	10	120	100	0.0508	[30]
CuFe_2_O_4_@C	0.1	30	20	90	97	0.038	[31]
MIL-88B@UiO-66	1	20	10	210	99.6	0.04845	[32]
β-FeOOH@GO	0.25	40	1.1	60	79.3	0.06322	[33]
MnFe_2_O_4_/g-C_3_N_4_	0.5	80	98	90	93.7	-	[34]
SiO_2_-Cu(II)-quinoline	0.2	5	88	150	95	0.0365	[35]
RGO/Fe_3_O_4_	0.25	20	10	120	96	-	[36]
HKUST-1/Fe_3_O_4_/CMF	0.3	10	120	240	98	-	[37]
GO/Cu_0.9_Fe_0.1_O	0.2	10	-	120	100	0.0378	[38]
GO-g-C_3_N_4_	1	10	-	240	67.3	0.00469	[39]
CuBTC-MA	0.1	10	30	60	97.8	0.07057	This work

## Data Availability

The data presented in this work are available in the article.

## Supplementary Materials

The following supporting information can be downloaded at: https://www.mdpi.com/article/10.3390/molecules30244691/s1, Figure S1: SEM images of CuBTC (A), MA-TMA (B), and CuBTC-MA composites (C). TEM image of CuBTC-MA composite (D). Elemental mapping of C, N, O, and Cu in CuBTC-MA composite (E); Figure S2: (A) Kubelka–Munk curves and (B) XPS valence band spectra of CuBTC and MA-TMA; Figure S3: (A) The degradation behaviors of MB by CuBTC-MA with different scavengers. (B) EPR spectra of DMPO-·OH in CuBTC-MA+H_2_O_2_ system. (C) PL spectra of TPA solution after 10 min of the photo-Fenton-like reaction by CuBTC-MA; Figure S4: (A) Chemical structure and surface electrostatic potential of MB and (B) HOMO-LUMO of MB; Figure S5: Possible photocatalytic degradation pathways of MB; Figure S6: Homemade photo-Fenton-like experimental device equipped with a 300 W halogen lamp (A) and the spectra of the lamp (B); Table S1: NPA charge distribution and Fukui indexes (ƒ) of MB.
