# Supplementary material for: Fabrication of Novel MOF/HOF Composite for Efficient Degradation of Methylene Blue via Photo-Fenton-like Process
Source: Molecules. 2025 Dec 8;30(24):4691. doi: 10.3390/molecules30244691 (PMC12735912; doi:10.3390/molecules30244691)
Supplement: Supplementary file 1 [file molecules-30-04691-s001.zip › molecules-3982207-supplementary.pdf]

Supporting Information

Fabrication of Novel MOF/HOF Composite for Efficient Degradation of Methylene Blue via Photo-Fenton-like Process

Yanfeng Zhang [1,†], Yong Huang [2,†], Han Leng [2] and Xuwei Chen [2,*]

[1] Intelligent Policing Key Laboratory of Sichuan Province, Sichuan Police College, Luzhou 646000, China; zhyf@scpolicec.edu.cn
[2] Department of Chemistry, College of Sciences, Northeastern University, Box 332, Shenyang 110819, China; chbu314@163.com (Y.H.); lengh@mails.neu.edu.cn (H.L.)
* Correspondence: chenxuwei@mail.neu.edu.cn; Tel.: +86-24-83684533; Fax: +86-24-83676698
† These authors contributed equally to this work.

Table of Contents

Figure S1. SEM images of CuBTC (A), MA-TMA (B), and CuBTC-MA composite (C). TEM image of CuBTC-MA composite (D). Elemental mapping of C, N, O and Cu in CuBTC-MA composite (E)..s3

Figure S2. (A) Kudelka-Munk curves and (B) XPS valence band spectra of CuBTC and MA-TMA.. ....s3

Figure S3 (A) The degradation behaviors of MB by CuBTC-MA with different scavengers. (B) EPR spectra of DMPO-·OH in CuBTC-MA+H2O2 system. (C) PL spectra of TPA solution after 10 min of the photo-Fenton-like reaction by CuBTC-MA......... ....s4

Figure S4. (A) Chemical structure and surface electrostatic potential of MB and (B) HOMO-LUMO of MB.. ..s4

Figure S5. Possible photocatalytic degradation pathways of MB...s5

Figure S6. Homemade photo-Fenton experimental device equipped with a 300 W halogen lamp lamp (A) and the spectra of the lamp(B)...................................。..s5

Table S1. NPA charge distribution and Fukui indexes (*f*) of MB..s6

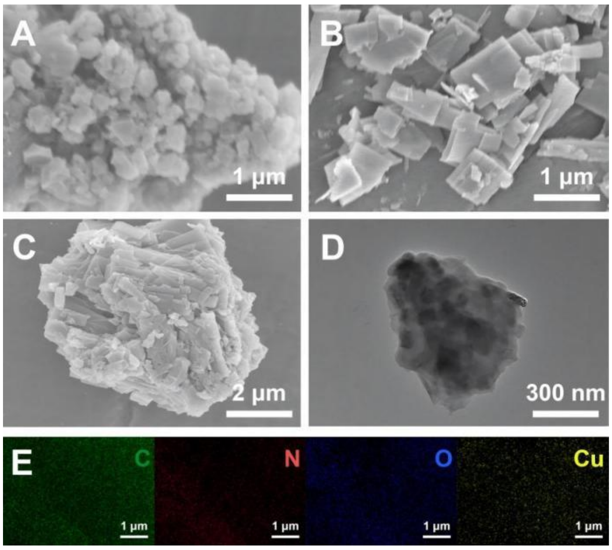

Figure S1. SEM images of CuBTC (A), MA-TMA (B), and CuBTC-MA composite (C). TEM image of CuBTC-MA composite (D). Elemental mapping of C, N, O and Cu in CuBTC-MA composite (E).

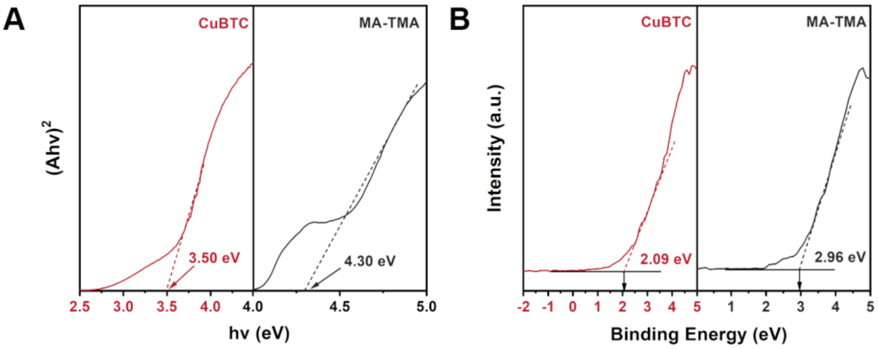

Figure S2. (A) Kudelka-Munk curves and (B) XPS valence band spectra of CuBTC and MA-TMA.

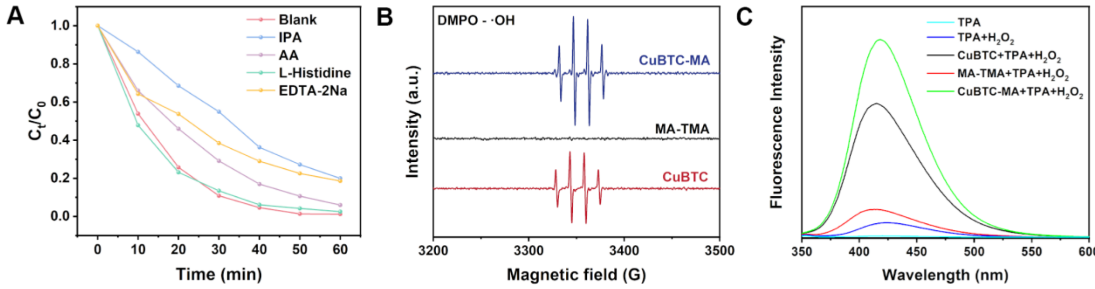

Figure S3. (A) The degradation behaviors of MB by CuBTC-MA with different scavengers. (B) EPR spectra of DMPO-·OH in CuBTC-MA+H_2O_2 system. (C) PL spectra of TPA solution after 10 min of the photo-fenton-like reaction by CuBTC-MA.

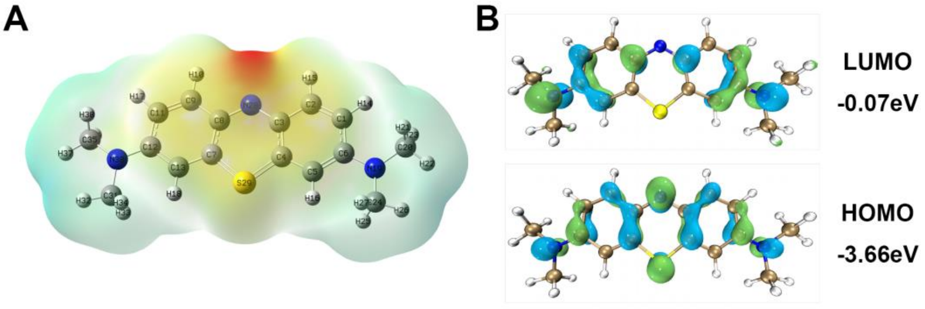

Figure S4. (A) Chemical structure and surface electrostatic potential of MB. (B) HOMO - LUMO of MB.

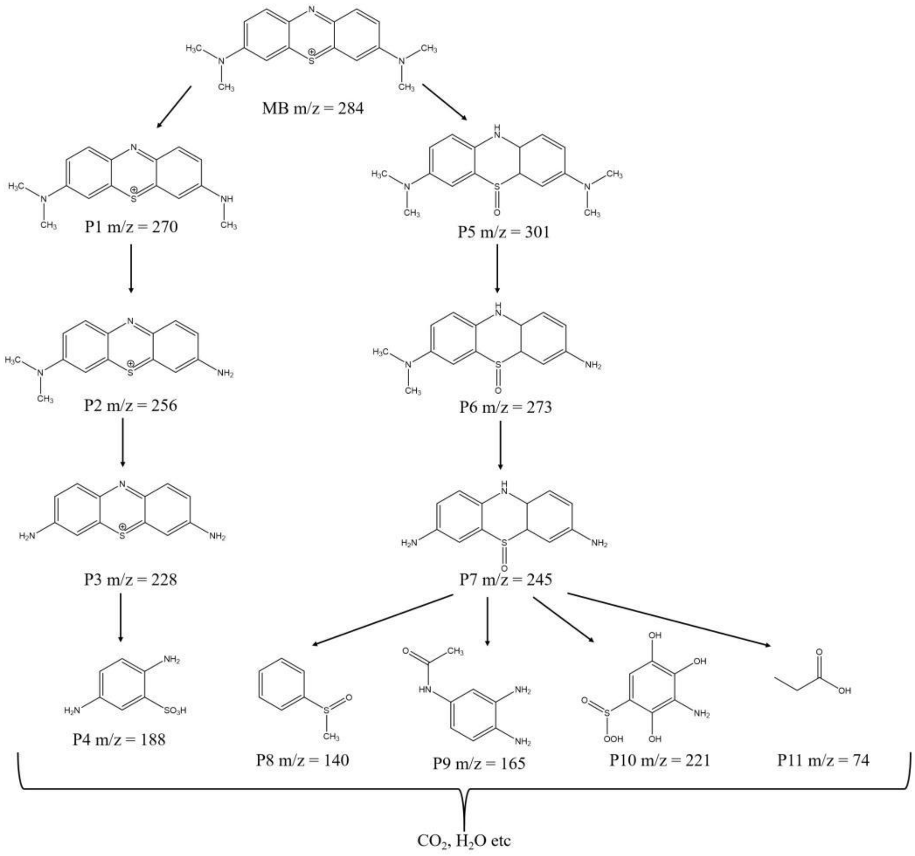

Figure S5. Possible photocatalytic degradation pathways of MB.

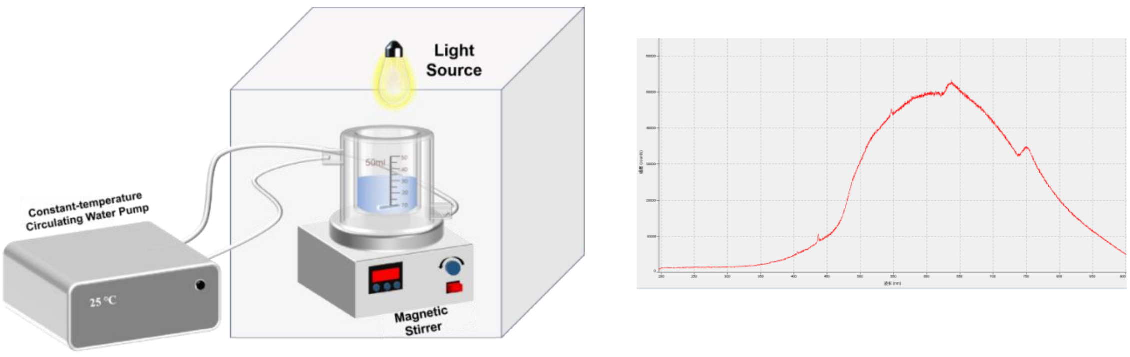

Figure S6 Homemade photo-Fenton experimental device equipped with a 300 W lamp (A) and the spectra of the lamp(B).

Table S1 NPA charge distribution and Fukui indexes (f) of MB.

Atom	Charge (0)	Charge (+1)	Charge (-1)	f^-	f^+	f^0	CDD
1(C)	-0.0353	-0.07	0.0032	0.0385	0.0347	0.0366	-0.0039
2(C)	-0.006	-0.0422	0.0162	0.0222	0.0362	0.0292	0.0141
3(C)	0.0442	0.0206	0.0863	0.0421	0.0235	0.0328	-0.0186
4(C)	0.0081	-0.0204	0.0185	0.0104	0.0285	0.0195	0.0182
5(C)	-0.0626	-0.0832	-0.0283	0.0343	0.0205	0.0274	-0.0138
6(C)	0.0821	0.0433	0.0986	0.0164	0.0388	0.0276	0.0224
7(C)	0.0081	-0.0205	0.0185	0.0104	0.0285	0.0195	0.0182
8(C)	0.0442	0.0206	0.0863	0.0421	0.0235	0.0328	-0.0186
9(C)	-0.006	-0.0422	0.0162	0.0222	0.0362	0.0292	0.0141
10(H)	0.0679	0.0453	0.0861	0.0182	0.0227	0.0204	0.0045
11(C)	-0.0353	-0.07	0.0032	0.0385	0.0347	0.0366	-0.0039
12(C)	0.0821	0.0433	0.0985	0.0164	0.0388	0.0276	0.0224
13(C)	-0.0626	-0.0832	-0.0283	0.0343	0.0205	0.0274	-0.0138
14(H)	0.0586	0.037	0.0776	0.019	0.0216	0.0203	0.0026
15(H)	0.0679	0.0453	0.0861	0.0182	0.0227	0.0204	0.0045
16(H)	0.0506	0.0341	0.0665	0.016	0.0165	0.0162	0.0005
17(H)	0.0586	0.037	0.0776	0.019	0.0216	0.0203	0.0026
18(H)	0.0506	0.0341	0.0665	0.016	0.0165	0.0162	0.0005
19(N)	-0.0118	-0.0535	0.069	0.0809	0.0416	0.0612	-0.0393
20(C)	-0.0151	-0.0279	0.004	0.0191	0.0128	0.0159	-0.0063
21(H)	0.0483	0.0308	0.075	0.0267	0.0175	0.0221	-0.0092
22(H)	0.0582	0.0412	0.0773	0.0191	0.017	0.0181	-0.0021
23(H)	0.0512	0.0369	0.0731	0.0219	0.0144	0.0181	-0.0075
24(C)	-0.0157	-0.0281	0.0029	0.0186	0.0125	0.0155	-0.0061
25(H)	0.0497	0.0369	0.0701	0.0204	0.0128	0.0166	-0.0076
26(H)	0.0586	0.0413	0.0779	0.0193	0.0173	0.0183	-0.002

27(H)	0.0472	0.0307	0.073	0.0258	0.0165	0.0211	-0.0093
28(N)	-0.0933	-0.1788	-0.0751	0.0182	0.0855	0.0519	0.0674
29(S)	0.1371	0.0335	0.1814	0.0443	0.1036	0.0739	0.0593
30(N)	-0.0118	-0.0534	0.069	0.0809	0.0416	0.0612	-0.0393
31(C)	-0.0157	-0.0281	0.0029	0.0186	0.0125	0.0155	-0.0061
32(H)	0.0586	0.0413	0.0779	0.0193	0.0173	0.0183	-0.002
33(H)	0.0472	0.0307	0.073	0.0258	0.0165	0.0211	-0.0093
34(H)	0.0497	0.0369	0.0701	0.0204	0.0128	0.0166	-0.0076
35(C)	-0.0151	-0.0279	0.004	0.0191	0.0128	0.0159	-0.0063
36(H)	0.0483	0.0308	0.075	0.0267	0.0175	0.0221	-0.0092
37(H)	0.0582	0.0412	0.0773	0.0191	0.017	0.0181	-0.0021
38(H)	0.0512	0.0369	0.0731	0.0219	0.0144	0.0181	-0.0075
